# Evaluation of next generation sequencing approaches for SARS-CoV-2

**DOI:** 10.1016/j.heliyon.2023.e21101

**Published:** 2023-10-19

**Authors:** Valentina Curini, Massimo Ancora, Lucija Jurisic, Valeria Di Lollo, Barbara Secondini, Luana Fiorella Mincarelli, Marialuigia Caporale, Ilaria Puglia, Luigina Di Gialleonardo, Iolanda Mangone, Marco Di Domenico, Adriano Di Pasquale, Alessio Lorusso, Maurilia Marcacci, Cesare Cammà

**Affiliations:** a Istituto Zooprofilattico Sperimentale dell’Abruzzo e del Molise, Teramo, Italy; b Faculty of Veterinary Medicine, University of Teramo, Teramo, Italy

**Keywords:** SARS-CoV-2, Whole genome sequencing, Methods, Comparison, Cost, Labor time

## Abstract

Within public health control strategies for SARS-CoV-2, whole genome sequencing (WGS) is essential for tracking viral spread and monitoring the emergence of variants which may impair the effectiveness of vaccines, diagnostic methods, and therapeutics. In this manuscript different strategies for SARS-CoV-2 WGS including metagenomic shotgun (SG), library enrichment by myBaits® Expert Virus-SARS-CoV-2 (Arbor Biosciences), nCoV-2019 sequencing protocol, ampliseq approach by Swift Amplicon® SARS-CoV-2 Panel kit (Swift Biosciences), and Illumina COVIDSeq Test (Illumina Inc.), were evaluated in order to identify the best approach in terms of results, labour, and costs. The analysis revealed that Illumina COVIDSeq Test (Illumina Inc.) is the best choice for a cost-effective, time-consuming production of consensus sequences.

## 1 Introduction

Severe acute respiratory syndrome coronavirus 2 (SARS-CoV-2) is a novel zoonotic coronavirus (family *Coronaviridae*, genus *Betacoronavirus*; subgenus *Sarbecovirus*, species *Severe acute respiratory syndrome-related coronavirus*) emerged in late 2019 in China [1, 2]and responsible for the COVID-19 (Coronavirus Disease 2019) respiratory disease pandemic.

Within public health COVID-19 control strategies, sequencing analysis is essential for tracking viral spread, monitoring the emergence of variants which may be associated with increased transmissibility or disease severity, or which may impair the effectiveness of vaccines, diagnostic methods, and therapeutics. Genomic surveillance of SARS-CoV-2 is currently performed using a combination of next generation sequencing (NGS) technologies and bioinformatics analysis [3]. In the last three years, millions of SARS-CoV-2 genomes have been sequenced worldwide and published in the publicly repositories including the Global Initiative on Sharing All Influenza Data (GISAID, https://www.gisaid.org/) [4].

The Istituto Zooprofilattico Sperimentale dell'Abruzzo e del Molise (IZSAM) supported the diagnostic workflow for COVID-19 in the Abruzzo region by testing thousands of human respiratory samples per day providing an excellent stand for investigating, by whole genome analysis, the local virus evolution, the origin of the occurring SARS-CoV-2 variants, and the genetic diversity of the circulating strains in the population [[5], [6], [7], [8], [9]].

Several NGS strategies for SARS-CoV-2 whole genome sequencing (WGS) have been developed [10]. The metagenomic shotgun (SG) approach was the method employed for the first SARS-CoV-2 sequencing from a broncho-alveolar lavage (BAL) sample in a patient with severe pneumonia in Wuhan [11] and then used for genomic surveillance activities during the early stages of the pandemic [3].

Unfortunately, this method lacks sensitivity and works efficiently when the abundance of the target virus is relatively high. So, in cases of clinical samples with low viral loads, a targeted sequencing approach could be ideal for obtaining the complete viral genome sequence. After a significant number of SARS-CoV-2 sequences became publicly available, some targeted methods including hybrid capture-based, amplicon-based, and ampliseq have been developed. Scrutiny, to increase the number of virus-related reads, the hybrid capture method can be used to enrich SARS-CoV-2 libraries by a mixture of virus-specific probes following library preparation. Alternatively, a specific set of primers can be used in the amplicon-based or ampliseq protocols. Indeed, several companies started rapidly the mass production of ampliseq kits to reduce time and cost of the library preparation sequencing workflow.

In this manuscript the performances of five different protocols for SARS-CoV-2 WGS such as metagenomic shotgun (SG) approach based on the sequence-independent single-primer amplification (SISPA) protocol, SARS-CoV-2 library enrichment by myBaits® Expert Virus-SARS-CoV-2 (Arbor Biosciences, Ann Arbor, MI USA), amplicon-based nCoV-2019 sequencing protocol (ARTIC) [12,13], and two ampliseq kit including Swift Amplicon® SARS-CoV-2 Panel kit (Swift Biosciences, Ann Arbor, MI USA) and Illumina COVIDSeq Test (Illumina Inc., San Diego, CA USA) were evaluated. These protocols were selected as, when the study was planned and designed, they were the first commercially available on the market in each different sequencing approach category. The SISPA method is our elected metagenomic SG protocol used in routine diagnostics. This protocol relies on a retro-transcription (RT) step followed by an amplification (PCR) step of total RNA using tagged-random primers [6]. The myBaits Expert Virus SARS-CoV-2 panel for SARS-CoV-2 library enrichment has been designed using all complete and partial genome sequences available in the NCBI database as of January 31, 2020. The flexible nature of hybridization capture allows the probes to enrich for even novel variants, point mutations and small or large insertions and deletions (indels). On January 22, 2020, within the ARTIC network, the first version of nCoV-2019 amplicon sequencing protocol was released [13]. This method comprises an RT step of RNA using random hexamers and an amplification step using two primers pools for a total of 98 SARS-CoV-2 specific primers pairs which produce amplicons of 400bp in length. These amplicons can be used as input of Illumina and MinION library kits. The Swift Amplicon® SARS-CoV-2 Panel kit (Swift Biosciences ) includes an RT step with random hexamers and an amplification step using tiled primer *ad hoc* designed on reference SARS-CoV-2 Wuhan-Hu-1 (NC_045512.2) and amplifying 341 amplicons of 116–255bp in lenght.

Finally, the Illumina COVIDSeq Test (Illumina Inc.) combines the nCoV-2019 multiplex PCR protocol (ARTIC) with the Illumina sequencing technology. This amplicon-based NGS test detects SARS-CoV-2 RNA in nasopharyngeal, oropharyngeal, and mid-turbinate nasal swabs and it is intended for detection of SARS-CoV-2 virus RNA in authorized countries (United States, Canada, Japan, Philippine, and South Africa) and virus genome analysis for research [[14], [15]].

The comparison described in this work was performed using three groups of samples named A, B and C characterized by different ranges of real time cycle threshold (Ct) values. Total raw reads, number of reads mapped onto Wuhan-Hu-1 SARS-CoV-2 reference genome (NC_045512.2), horizontal and vertical coverage (Hcov and Vcov), and variant calling were evaluated for each protocol as well as working time and costs per sample.

## 2 Materials and methods

### Ethical statement

The results analyzed in the present study derive from the official control activities performed by the Public Health Local Authorities and no ethical approval was specifically requested.

### 2.1 SARS-CoV-2 positive samples datasets

Nasopharyngeal swabs samples were collected from April 2020 to April 2021 from individuals which were either hospitalized, selected because of their contact history with infected individuals, or included into the study in the framework of the screening programs for workers of the National Health System. Samples were collected in the hospitals of different cities of Abruzzo region including Teramo (Ospedale Giuseppe Mazzini), Atri (Ospedale Civile S. Liberatore), Pescara (Presidio Ospedaliero Santo Spirito), Avezzano (Ospedale SS Nicola e Filippo) and L'Aquila (Ospedale Regionale S. Salvatore). All samples were tested by TaqPath™ COVID-19 CE-IVD RT-PCR Kit (Thermofisher scientific, Waltham, MA USA) as described previously by our group [6]. The results of the real time RT-PCR test are expressed as cycle threshold (Ct) values that represent the cycle number at which the fluorescence generated within a reaction crosses the fluorescence threshold. At the threshold cycle, a detectable amount of amplicon product has been generated during the early exponential phase of the reaction. The threshold cycle is inversely proportional to the original quantity of the target. For practical reasons, as the adopted molecular tests binds to three different viral genome targets, only values for the N protein encoding gene were taken into consideration.

### 2.2 Sequencing protocols

The comparison was performed using three different sets of SARS-CoV-2 positive nasopharyngeal swabs including: group A, consisting of 11 samples with high viral load and cycle threshold (Ct) range of 16–25 processed by the i) SISPA protocol; ii) SISPA with myBaits Expert Virus SARS-CoV-2 panel (Arbor Biosciences) and iii) nCoV-2019 sequencing protocol (Table 1); group B, consisting of 11 samples with low viral load and Ct range of 23–34 processed by the i) nCoV-2019 sequencing protocol and ii) Swift Amplicon® SARS-CoV-2 Panel kit (Swift Biosciences) (Table 2); group C, consisting of 10 samples with a wider Ct range of 17–28 processed by the Illumina COVIDSeq Test (Illumina Inc.) (Table 3).

**Table 1:** Samples of group A processed using the SISPA protocol, the SISPA + myBaits® SARS-CoV-2 panel, and the nCoV-2019 sequencing protocol. The total number of raw reads and the number of reads mapped to SARS-CoV-2 reference genome (NC_045512) are shown.

		SISPA protocol		SISPA + myBaits®		nCoV-2019 protocol	
SAMPLE	Ct value	Total raw reads	SARS-CoV-2 reads	Total raw reads	SARS-CoV-2 reads	Total raw reads	SARS-CoV-2 reads
**1A**	25	1203226	114	347128	43388	1450538	163966
**2A**	25	914890	93	429444	65655	552344	69233
**3A**	25	1051024	1796	1553764	514658	1402108	249371
**4A**	23	582276	710	714620	224740	728388	160780
**5A**	22	707544	2215	1860880	579876	786298	169107
**6A**	19	993018	87143			1664668	425561
**7A**	23	1154510	83	525678	32099	670354	82981
**8A**	23	922546	2250	2285742	647067	612772	141952
**9A**	16	1483578	397940			1193544	482387
**10A**	17	1051604	91172			952890	345494
**11A**	16	754600	34023			504736	169564

**Table 2:** Samples of group B processed using the Swift Amplicon® SARS-CoV-2 Panel kit and the nCoV-2019 sequencing protocol. The total number of raw reads and the number of reads mapped to SARS-CoV-2 reference genome (NC_045512) are shown.

		Swift Amplicon® SARS-CoV-2 Panel kit		nCoV-2019 protocol	
SAMPLE	Ct value	Total raw reads	SARS-CoV-2 reads	Total raw reads	SARS-CoV-2 reads
**1B**	23	7672958	12116	2211146	603504
**2B**	32	41796	371	254380	48591
**3B**	33	469244	326	285596	46973
**4B**	31	36160	328	174658	21945
**5B**	31	1372578	4906	1871720	469665
**6B**	28	302422	3518	3488984	769230
**7B**	34	57054	476	642330	190066
**8B**	33	180218	317	106156	27108
**9B**	27	251172	2193	2251204	546295
**10B**	30	402088	2795	2460972	556003
**11B**	25	1752374	7265	1813082	525434

**Table 3:** Samples of group C processed by the Illumina COVIDSeq Test. The total number of raw reads and the number of reads mapped to SARS-CoV-2 reference genome (NC_045512) are shown.

SAMPLE	Ct value	Totale Raw Reads	SARS-CoV-2 reads
**1C**	18	5849008	1325271
**2C**	18	6444098	1394975
**3C**	17	4775402	1133143
**4C**	17	4949636	1151904
**5C**	18	5310330	1214837
**6C**	20	4754340	1121335
**7C**	23	4530674	1048553
**8C**	25	4742530	1024947
**9C**	24	2858264	833627
**10C**	28	2214970	457572

Unfortunately, we were not able to test the same set of samples with all WGS approaches as for the fast turnaround of samples during the early phases of COVID-19 at IZSAM.

For group A (Table 1), total RNA was used for the assessment of the SISPA protocol [6]. After TURBO DNase (Thermo Fisher Scientific, Waltham, MA USA) treatment and purification by RNA Clean and Concentrator-5 Kit (Zymo Research, Irvine, CA USA), RNA was retro-transcribed to cDNA using SuperScript® IV Reverse Transcriptase (Thermo Fisher Scientific, Waltham, MA USA) and two primers including the random-tagged primer FR26RV-N 5′-GCCGGAGCTCTGCAGATATCNNNNNN-3′ and a poly-A tagged primer FR40RV-T 5′-GCCGGAGCTCTGCAGATATCTTTTTTTTTTTTTTTTTTTT-3′, and then amplified with the primer-tag FR20RV 5′-GCCGGAGCTCTGCAGATATC-3′. The SISPA products were then employed for library preparation using Illumina® DNA Prep, (M) Tagmentation (96 Samples) (Illumina Inc.) according to the manufacturer's protocol.

A subset of group A consisting of 7 sample libraries (1A–5A, 7A–8A) was enriched with myBaits Expert Virus SARS-CoV-2 panel (Arbor Biosciences) following the manufacturer's instructions. As myBaits® system is not compatible with Illumina® DNA Prep, (M) Tagmentation kit, an initial pre-treatment was required to deplete the residual streptavidin-affinity molecules. Illumina libraries were mixed with various blocking nucleic acids, denatured, and then combined with other hybridization reagents (including baits). These hybridization reactions were incubated for 16 h to allow baits to encounter and hybridize with SARS-CoV-2 library molecules. Following capture clean-up, bead-bound enriched libraries were amplified with universal P5/P7 primers for 14 cycles using the KAPA HiFi HotStart polymerase system and purified with Expin™ PCR SV (GeneAll, Seoul, Korea).

Group A sample set was also processed by the nCoV-2019 sequencing protocol. Total RNA was reverse-transcribed by random hexamers (Thermo Fisher Scientific, Waltham, MA USA) and cDNA was amplified with two separate primers pools specific for SARS-CoV-2 genome. Obtained amplicons were processed for library preparation using Illumina® DNA Prep, (M) Tagmentation (96 Samples) (Illumina Inc.) according to the manufacturer's protocol.

Group B samples were sequenced using two targeted approaches including the nCoV-2019 sequencing protocol and the Swift Amplicon® SARS-CoV-2 Panel kit (Swift Biosciences) according to the manufacturer's protocol. This protocol consists of an RT step using random hexamers, a direct cDNA amplification by a primer mix of Swift Amplicon SARS-CoV-2 Research Panel, and an incubation with Indexing Reaction Mix to create the libraries.

Finally, samples of group C were processed by the Illumina COVIDSeq Test (Illumina Inc.), according to the manufacturer's instructions. This protocol is characterized by RNA-to-cDNA conversion, cDNA amplification with the 98 2019-nCoV primers couples and library preparation [[15], [16]]. Group A, B and C sample libraries were sequenced onto MiniSeq platform (Illumina Inc.) using the MiniSeq Mid Output Kit (300-cycles) and standard 150 bp paired-end reads.

### 2.3 SARS-CoV-2 bioinformatics workflow

A pipeline dedicated to SARS-CoV-2 [17] was implemented in GENPAT, the bioinformatic repository and platform (https://genpat.izs.it/cmdbuild/ui/#login) of the *"National Reference Centre for Whole Genome Sequencing of microbial pathogens: database and bioinformatic analysis"* (GENPAT). Raw reads were trimmed using Trimmomatic (version 0.36, parameters: illuminaclip:2:30:10, leading:25 trailing:25 sliding windows:20:25, minlen: 36) [18] and then mapped to Wuhan-Hu-1 reference genome (NC_045512.2) using Snippy (version 4.5.1, default parameters) (https://github.com/tseemann/snippy). Consensus sequences were generated using iVar (version 1.3, parameters: minimum length of read to retain after trimming m = 1, minimum quality threshold for sliding window to pass q = 20) [19]. The lineage was assigned using the algorithm pangoLEARN from the workflow PANGOLIN 2.0 (https://github.com/cov-lineages/pangolin). Nucleotide positions were calculated using Wuhan-Hu-1 (NC_045512.2) as reference genome. The horizontal and vertical coverage (Hcov and Vcov) values of the consensus sequences were calculated. For clarity, Hcov is the length of the consensus sequence that cover the reference used for mapping and is expressed in percentage (e.g. Hcov 100 %). Vcov is the average number of sequenced bases that align to known reference bases and is expressed as depth of sequencing (e.g. 50X)

### 2.4 Statistical analysis

Total raw reads and SARS-CoV-2 mapped reads obtained from group A samples by SISPA, SISPA + myBaits® Expert Virus SARS-CoV-2 panel (Arbor Biosciences), and nCoV-2019 sequencing protocol were compared by using the Kruskal-Wallis Test. Total raw reads and SARS-CoV-2 mapped reads obtained from group B samples by nCoV-2019 sequencing protocol and the Swift Amplicon® SARS-CoV-2 Panel kit (Swift Biosciences) were compared by using the Mann-Whitney *U* test calculator for two independent set of samples. Differences were considered statistically significant when p < 0.05.

### 2.5 Working time and cost calculations

Working time and cost were calculated taking into account a set of 96 samples, after RNA extraction. For working time, the duration of each method was calculated excluding the sequencing duration time which was the same for all protocols. Cost per sample was calculated considering only reagents and commercial kits employed for RNA manipulation and library preparation. The costs of consumables, equipment maintenance assistance, and labor were excluded.

## 3 Results

### 3.1 The Illumina COVIDseq Test produced SARS-CoV-2 consensus sequences with the highest horizontal and vertical coverages

**Fig. 1:**
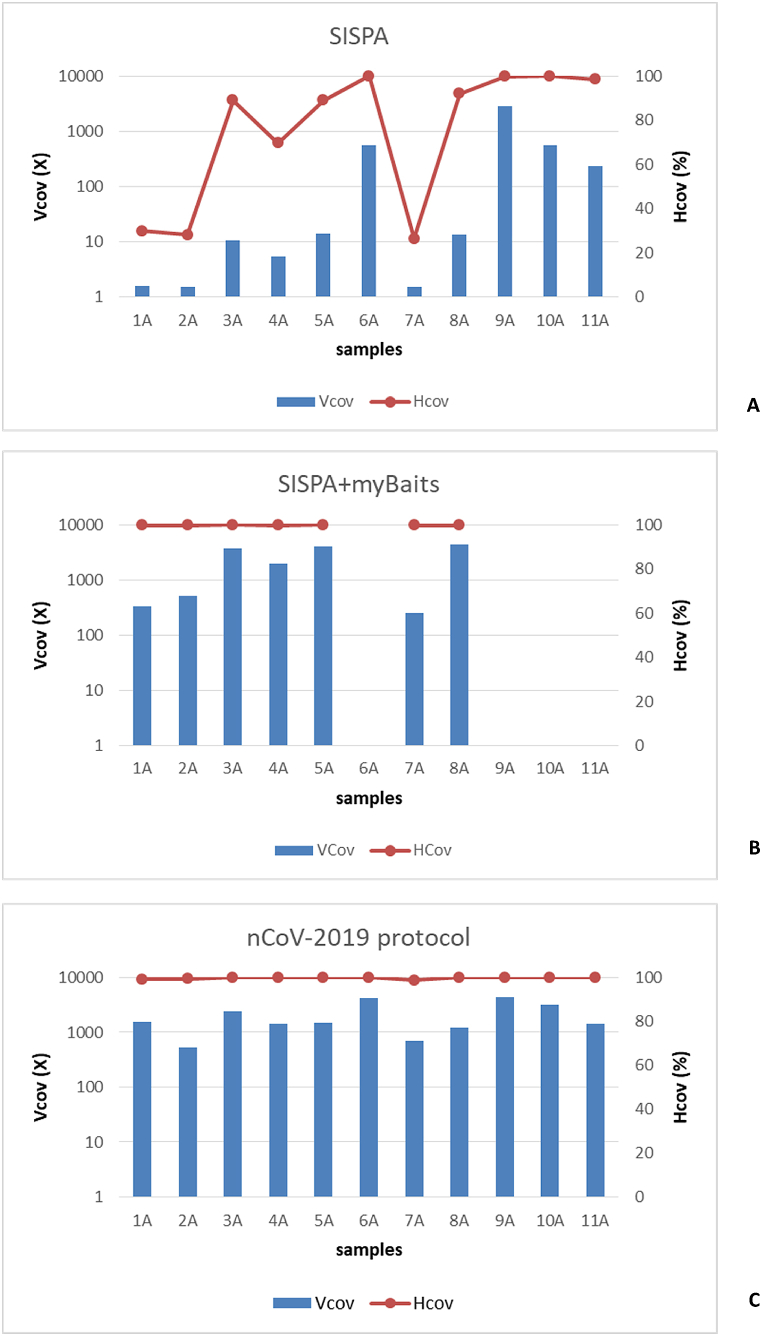
Vertical (Vcov) and horizontal coverage (Hcov%) obtained from samples of Group A.

The group A sample set (11 samples, Ct range 16–25) was processed using the SISPA protocol, the SISPA + myBaits® Expert Virus SARS-CoV-2 panel (Arbor Biosciences), and the nCoV-2019 sequencing protocol. For the three approaches the number of total raw reads produced was overall similar (p = 0.86), while significant differences in terms of reads mapping to SARS-CoV-2 reference genome (NC_045512.2) were observed. Indeed, the number of SARS-CoV-2 reads obtained by SISPA was lower than of those obtained by SISPA + myBaits® Expert Virus SARS-CoV-2 panel and nCoV-2019 sequencing protocol (p = 0.003, Table 1). SISPA gave consensus sequences with 99 % Hcov and Vcov >200X for four samples (Ct 16–19), while from samples with Ct 22–25, SISPA gave only partial sequences (Hcov 28–92 %, Vcov 1,5-14X, Fig. 1A). By contrast, nCoV-2019 sequencing protocol and SISPA + myBaits® Expert Virus SARS-CoV-2 panel approach generated consensus sequence with Hcov ≥99 % for all samples and Vcov >253X (Fig. 1B and C) without any statistically significant difference in terms of number of SARS-CoV-2 mapping reads (p > 0.05). Moreover, all the consensus sequences obtained by these three methods were compared to assess their accuracy. All the consensus sequences were identical, except for three samples processed by the SISPA + myBaits® Expert Virus SARS-CoV-2 panel approach that showed IUPAC nt in some positions (sample 1A, position 27046; sample 2A, position 17427; sample 7A, positions 830, 28881, 28882, 28883).

**Fig. 2:**
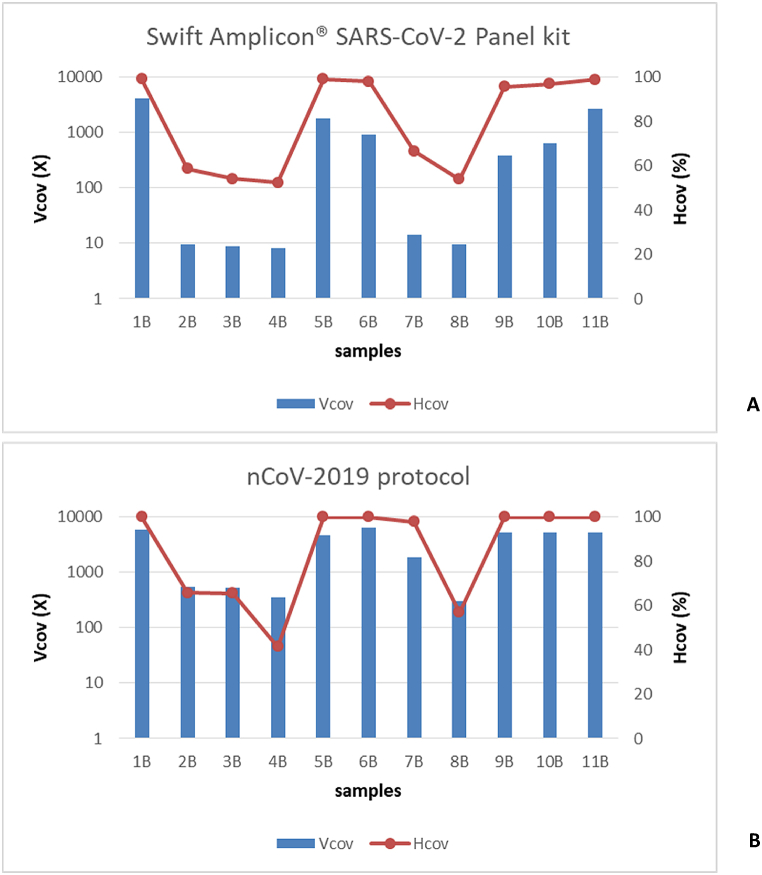
Vertical (Vcov) and horizontal coverage (Hcov%) obtained for group B samples.

Samples of group B (11 samples, Ct 23–34) were processed by using the nCoV-2019 sequencing protocol and the Swift Amplicon® SARS-CoV-2 Panel kit. Both methods produced a similar mean number of total raw reads (p = 0.13104, Table 2) while some differences between two methods were found in terms of number of SARS-CoV-2 mapping reads (p = 0.00341). The nCoV-2019 protocol produced consensus sequences with Hcov >99 % and Vcov >4.500X for 6 out of 11 samples (Ct 23–31); for the remaining 5 samples with Ct 31–34 the Hcov was 41–97 % with Vcov 301-1808X (Fig. 2B). Conversely, the Swift protocol generated consensus sequence with Hcov of 98–99 % for 4 samples with Ct of 23, 25, 28 and 31 with Vcov >900X; 2 samples (Ct 27 and 30) with Hcov of 96 % and Vcov >382X; 5 samples (Ct 31–34) with low Hcov (52–66 %) and Vcov (8-14X) (Table 2, Fig. 2A). Moreover, the consensus sequences obtained by the Swift protocol showed two small gaps (7694–7700 nt and 20576–20609 nt) and some degenerate bases at positions 241 and 14448.

**Fig. 3:**
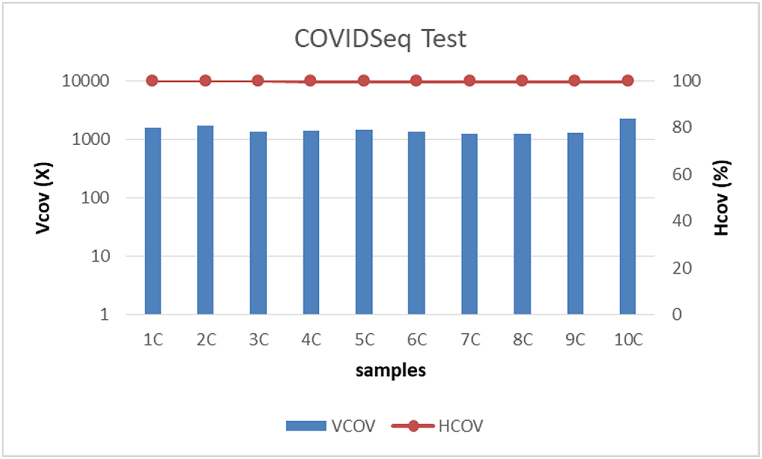
Vertical (Vcov) and horizontal coverage (Hcov%) obtained for group C samples.

The group C (10 samples, Ct 17–28), processed with the Illumina COVIDSeq Test, produced the highest number of reads mapped to SARS-CoV-2 mapping reads (Table 3). All samples showed a mean Hcov of 99.8 % and a mean Vcov of 1425X (Fig. 3).

### 3.2 Working time and cost per sample

**Fig. 4:**
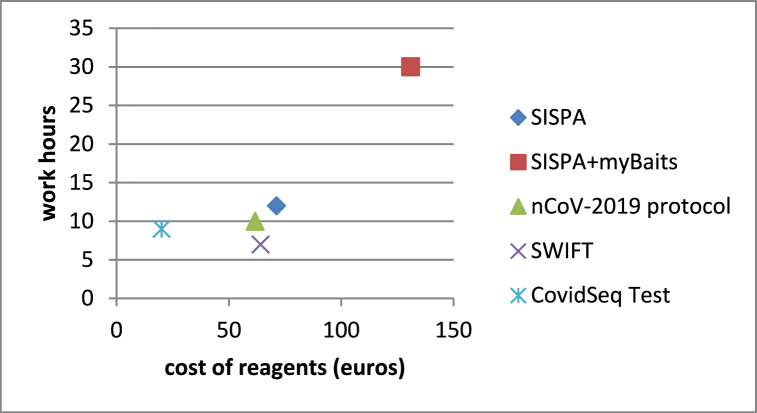
**Cost of reagents and work hours for each protocol**.

For a set of 96 samples, the SISPA protocol workflow takes 2 working days in total, from RNA manipulation to library preparation and the cost was approximately 71 euros per sample. SISPA + myBaits® Expert Virus SARS-CoV-2 panel protocol required instead 3 working days (including 16h of hybridization) and 131 euros per sample (71 euros for SISPA and 60 euros for enrichment). The nCoV-2019 sequencing protocol takes 2 working days and 62 euros per sample while the Swift Amplicon® SARS-CoV-2 Panel kit takes 7 working hours and 64 euros per sample. Finally, the Illumina COVIDSeq Test required 9 working hours and costs 20 euros per sample (Fig. 4). For all methods, the MiniSeq Mid Output Kit (300-cycles) cartridge was used with standard 150 bp paired-end reads, resulting in a sequencing duration time of 20 h.

## 4 Discussion

In this work, five protocols for SARS-CoV-2 WGS were compared. The SISPA protocol produced nearly complete consensus sequence only from 4 samples with Ct < 20 with Vcov >200X; from samples with Ct > 20 only partial genome sequences were obtained. These results highlighted the benefits and drawbacks of this “universal” protocol based on random primers able to anneal all RNA molecules present in a sample. Ideally, the SISPA method should be used at the beginning of a new outbreak as first-line approach to identify unknown or unexpected pathogens [20]. In our laboratory settings, the SISPA protocol was adopted for metagenomics SG approach to reveal genome constellations of segmented RNA viruses as Bluetongue virus (BTV) [[21], [22], [23]], to identify novel atypical BTV serotypes [24,25], and to obtain complete or nearly complete genome sequences of different RNA viruses [[26], [27], [28]] including Rift Valley fever virus (RVFV) [29] and Crimean and Congo Haemorrhagic Fever virus (CCHFV) [30], but it lacks in sensitivity when the viral load is low.

To improve the sensitivity of the SISPA protocol on samples with suboptimal viral load, the enrichment of libraries by myBaits® Expert Virus SARS-CoV-2 panel was implemented. Complete consensus sequences with high Hcov and Vcov values were indeed obtained from 7 samples whose sequencing failed by using only the SISPA protocol. This can be reasonably explained by the removal of non-target reads followed by the enrichment of SARS-CoV-2 reads by the probes binding to the beads. However, the combination of SISPA + myBaits® Expert Virus SARS-CoV-2 panel is expensive (131 euros per sample) and laborious (3 working days).

The nCoV-2019 sequencing protocol (ARTIC) showed similar performances of the SISPA + myBaits® Expert Virus SARS-CoV-2 panel but it is more convenient in terms of working time and cost.

The Swift protocol was more cost-effective and less time-consuming than nCoV-2019 sequencing protocol, but it produced for all samples consensus sequences with lower Hcov and Vcov values with two gaps of 7 and 34 nt in positions 7694 and 20576, respectively and some IUPAC in positions 241 and 14448 which are not present in the homologues nCoV-2019 consensus sequences. We believe that the observed gaps and IUPAC were likely due to the low amplification efficiency of the primers designed on these regions and to the low Vcov, respectively.

Finally, the Illumina COVIDSeq Test showed the highest number of SARS-CoV-2 mapped reads with high values of Vcov and Hcov of the consensus sequences produced. This approach was demonstrated to be efficient regardless the viral load (Ct = 28). This aspect combined with time (9 h) and cost (20 euros per samples) makes the Illumina COVIDSeq Test the first choice for SARS-CoV-2 WGS. Recently, in our laboratory settings, the Illumina COVIDSeq Test protocol has been automated on the liquid handling station Microlab Star (Hamilton, Reno, NE USA), by standardizing every step of the library preparation workflow. The automation of the Illumina COVIDSeq Test libraries preparation significantly reduced the human error and increased the reproducibility of results [31].

This study has certainly some pitfalls. First, we did not test the same set of samples for all WGS approaches as for the fast turnaround of samples during the early phases of COVID-19 at IZSAM. Second, the samples employed for the comparison were collected during the first and second waves of COVID-19 in Italy (up to April 2021) thus variants of concern (VOCs) which emerged later such as the Beta, Gamma, Omicron and related sub-lineages were not included. However, if on the one hand this lack hampered a proper comparison with a heterogeneous set of SARS-CoV-2 variants, on the other, all WGS analysis conducted at IZSAM from April 2021 onward, were performed, efficiently, by using only the Illumina COVIDSeq Test protocol which was demonstrated to be by far the best approach for SARS-CoV-2 WGS also by other research groups worldwide [[14], [15]]. Moreover, recently, the Illumina COVIDSeq Test (Illumina Inc.) has been validated as diagnostic test to detect SARS-CoV-2 variants and to monitor their evolution [32].

In conclusion, the Illumina COVIDSeq Test protocol is certainly the best choice for a cost-effective and time-consuming approach for SARS-CoV-2 sequencing. Accordingly, similar strategies should be adopted for other viruses of public health importance, which require systematic surveillance and monitoring.

## Author contribution statement

Valentina Curini, Maurilia Marcacci: Conceived and designed the experiments; Performed the experiments; Analyzed and interpreted the data; Wrote the paper.

Massimo Ancora, Marialuigia Caporale, Ilaria Puglia: Performed the experiments; Analyzed and interpreted the data.

Lucija Jurisic, Valeria Di Lollo, Barbara Secondini, Luana Fiorella Mincarelli, Luigina Di Gialleonardo, Marco Di Domenico: Performed the experiments.

Iolanda Mangone, Adriano Di Pasquale: Analyzed and interpreted the data.

Alessio Lorusso: Conceived and designed the experiments; Analyzed and interpreted the data; Contributed reagents, materials, analysis tools or data; Wrote the paper.

Cesare Cammà: Conceived and designed the experiments; Contributed reagents, materials, analysis tools or data.

## Data availability statement

Sequence data associatedwith this study has been deposited at https://gisaid.org/EpiCoV™ database under the following accession number: group A samples: EPI_ISL_436718, EPI_ISL_436719, EPI_ISL_436720, EPI_ISL_436721, EPI_ISL_436722, EPI_ISL_429228, EPI_ISL_436723, EPI_ISL_436724, EPI_ISL_429226, EPI_ISL_429227, EPI_ISL_429229; group B samples: EPI_ISL_436725, EPI_ISL_436726, EPI_ISL_436727, EPI_ISL_436728, EPI_ISL_436729, EPI_ISL_436731, EPI_ISL_436732; group C samples: EPI_ISL_1707607, EPI_ISL_1788750, EPI_ISL_1707606, EPI_ISL_1707616, EPI_ISL_1707615, EPI_ISL_1707605, EPI_ISL_1707628, EPI_ISL_1707632, EPI_ISL_1707635, EPI_ISL_2921241. The sequences of the same viral strains obtained by means of different NGS approaches data are available upon request.

## Funding

This work was supported by funding from the European Union's Horizon 2020 Research and Innovation programme (*One Health European Joint Programm*e under grant agreement No 773830, recipient Alessio Lorusso), from the 10.13039/501100003196Ministry of Health, Italy Ricerca Corrente 2020(*PanCO “Epidemiologia e Patogenesi dei coronavirus umani ed animali*”, recipient Alessio Lorusso), and Ricerca Strategica 2020 (“*Suscettibilità dei mammiferi a SARS-COV-2: rischi di zoonosi inversa e possibilità in medicina traslazionale*”, recipient Alessio Lorusso) and it was partially supported by EU funding within the NextGenerationEU-MUR PNRR Extended Partnership initiative on Emerging Infectious Diseases (Project no. PE00000007, INF-ACT).

## Declaration of competing interest

The authors declare that they have no known competing financial interests or personal relationships that could have appeared to influence the work reported in this paper.
